# Renin Angiotensin System Blockers and Risk of Mortality in Hypertensive Patients Hospitalized for COVID-19: An Italian Registry

**DOI:** 10.3390/jcdd9010015

**Published:** 2022-01-06

**Authors:** Fabio Angeli, Paolo Verdecchia, Antonella Balestrino, Claudio Bruschi, Piero Ceriana, Luca Chiovato, Laura Adelaide Dalla Vecchia, Francesco Fanfulla, Maria Teresa La Rovere, Francesca Perego, Simonetta Scalvini, Antonio Spanevello, Egidio Traversi, Dina Visca, Michele Vitacca, Tiziana Bachetti

**Affiliations:** 1 Department of Medicine and Surgery, University of Insubria, 21100 Varese, Italy; Antonio.spanevello@icsmaugeri.it (A.S.); dina.visca@icsmaugeri.it (D.V.); 2 Department of Medicine and Cardiopulmonary Rehabilitation, Istituti Clinici Scientifici Maugeri IRCCS, 21049 Tradate, Italy; 3 Fondazione Umbra Cuore e Ipertensione-ONLUS and Division of Cardiology, Hospital S. Maria Della Misericordia, 06100 Perugia, Italy; verdecchiapaolo@gmail.com; 4 Department of Pulmonary Rehabilitation, Istituti Clinici Scientifici Maugeri IRCCS, 27100 Pavia, Italy; antonella.balestrino@icsmaugeri.it (A.B.); piero.ceriana@icsmaugeri.it (P.C.); francesco.fanfulla@icsmaugeri.it (F.F.); 5 Department of Pulmonary Rehabilitation, Istituti Clinici Scientifici Maugeri IRCCS, 27040 Montescano, Italy; claudio.bruschi@icsmaugeri.it; 6 Unit of Internal Medicine and Endocrinology, Laboratory for Endocrine Disruptors, Istituti Clinici Scientifici Maugeri IRCCS, 27100 Pavia, Italy; luca.chiovato@icsmaugeri.it; 7 Department of Internal Medicine and Therapeutics, University of Pavia, 27100 Pavia, Italy; 8 Department of Cardiac Rehabilitation, Istituti Clinici Scientifici Maugeri IRCCS, 20019 Milano, Italy; laura.dallavecchia@icsmaugeri.it; 9 Department of Cardiac Rehabilitation, Istituti Clinici Scientifici Maugeri IRCCS, 27040 Montescano, Italy; mariateresa.larovere@icsmaugeri.it (M.T.L.R.); egidio.traversi@icsmaugeri.it (E.T.); 10 Department of Subacute Therapy, Istituti Clinici Scientifici Maugeri IRCCS, 20019 Milano, Italy; francesca.perego@icsmaugeri.it; 11 Department of Cardiac Rehabilitation, Istituti Clinici Scientifici Maugeri IRCCS, 25065 Lumezzane, Italy; simonetta.scalvini@icsmaugeri.it; 12 Department of Pulmonary Rehabilitation, Istituti Clinici Scientifici Maugeri IRCCS, 25065 Lumezzane, Italy; michele.vitacca@icsmaugeri.it; 13 Scientific Direction, Istituti Clinici Scientifici Maugeri IRCCS, 27100 Pavia, Italy; tiziana.bachetti@icsmaugeri.it

**Keywords:** SARS-CoV-2, COVID-19, renin–angiotensin system, ACE2, ACE inhibitors, angiotensin receptor blockers, angiotensin-converting enzyme inhibitors

## Abstract

Background: It is uncertain whether exposure to renin–angiotensin system (RAS) modifiers affects the severity of the new coronavirus disease 2019 (COVID-19) because most of the available studies are retrospective. Methods: We tested the prognostic value of exposure to RAS modifiers (either angiotensin-converting enzyme inhibitors [ACE-Is] or angiotensin receptor blockers [ARBs]) in a prospective study of hypertensive patients with COVID-19. We analyzed data from 566 patients (mean age 75 years, 54% males, 162 ACE-Is users, and 147 ARBs users) hospitalized in five Italian hospitals. The study used systematic prospective data collection according to a pre-specified protocol. All-cause mortality during hospitalization was the primary outcome. Results: Sixty-six patients died during hospitalization. Exposure to RAS modifiers was associated with a significant reduction in the risk of in-hospital mortality when compared to other BP-lowering strategies (odds ratio [OR]: 0.54, 95% confidence interval [CI]: 0.32 to 0.90, *p* = 0.019). Exposure to ACE-Is was not significantly associated with a reduced risk of in-hospital mortality when compared with patients not treated with RAS modifiers (OR: 0.66, 95% CI: 0.36 to 1.20, *p* = 0.172). Conversely, ARBs users showed a 59% lower risk of death (OR: 0.41, 95% CI: 0.20 to 0.84, *p* = 0.016) even after allowance for several prognostic markers, including age, oxygen saturation, occurrence of severe hypotension during hospitalization, and lymphocyte count (adjusted OR: 0.37, 95% CI: 0.17 to 0.80, *p* = 0.012). The discontinuation of RAS modifiers during hospitalization did not exert a significant effect (*p* = 0.515). Conclusions: This prospective study indicates that exposure to ARBs reduces mortality in hospitalized patients with COVID-19.

## 1. Introduction

At the beginning of the severe acute respiratory syndrome coronavirus 2 (SARS-CoV-2) pandemic, the evidence that angiotensin-converting enzyme 2 (ACE2) acts as the functional receptor for the spike glycoprotein of the virus generated some concerns regarding the potential deleterious effect of renin–angiotensin system (RAS) modifiers, which had been shown to increase ACE2 expression in some experimental models [1].

With advancing knowledge on the role of RAS in the pathogenesis of the new coronavirus disease 2019 (COVID-19), academic literature recognized that, while it has been coopted as the entry point for the SARS-CoV-2 virus on host cells, the ACE2 enzyme also modulates the balance between vasoconstrictors and vasodilators within the heart and kidney, and it plays a significant role in regulating cardiovascular and renal functions [2,3,4].

Several observational studies conducted to clarify this controversial issue generated mixed results [4]. The majority of the available studies did not show any sign of harm associated with ACE inhibitors (ACE-Is) or angiotensin receptor blockers (ARBs) in patients with COVID-19 (no significant association between the chronic use of RAS modifiers and either the risk to contract an infection or the risk of developing a severe or lethal form of the disease) [4,5]. Conversely, some retrospective clinical studies demonstrated a lower risk of in-hospital death among patients taking ACE-Is or ARBs than among patients not receiving these drugs [6,7].

We designed a prospective study in a cohort of hypertensive patients hospitalized for COVID-19 in order to specifically evaluate the prognostic impact of antihypertensive medications, including RAS modifiers.

## 2. Materials and Methods

We analyzed data from patients in 5 hospitals of the Lombardy region and belonging to the Maugeri Care and Research Institutes Network. The protocol was approved by the Ethical Committee of our institution, and patients gave their written informed consent to participate.

Details of the protocol have been published [8,9]. Briefly, our study was a pre-designed registry of patients hospitalized for COVID-19 with subsequent prospective collection of data; the primary study outcome was all-cause mortality during hospitalization. Secondary outcomes included death and new hospitalization after 2 years from COVID-19 recovery (follow-up is still ongoing).

Diagnosis of viral infection was confirmed in all patients by using RNA reverse transcription–polymerase chain reaction (RT-PCR) assays from nasopharyngeal swab specimens [10]. Demographic, laboratory, and clinical management data were collected at admission and throughout the entire in-hospital stay. Laboratory parameters were assessed using standard techniques. PaO_2_/FIO_2_ ratio was used to estimate the severity of respiratory dysfunction, and high-sensitivity cardiac troponin was used as a marker of myocardial injury (as documented by troponin elevation > 5 pg/mL, according to the reference values of our laboratory).

The presence of comorbidities was defined according to documented medical history, as collected by investigators at study site level, including examination of electronic health record data of the Lombardy region. All clinical evaluations were performed by attending physicians during the clinical interview and through examination of medical records. Comorbidities (including type II diabetes, chronic kidney disease, dyslipidemia, hypertension, previous cardiac events) were defined according to current guidelines [11,12,13,14]. Previous cardiac events included history of heart failure (symptomatic syndrome, as graded according to the New York Heart Association functional classification or prior hospitalization for acute heart failure requiring intravenous therapy) and coronary artery disease (as defined by at least one of the following criteria: (1) presence of any epicardial coronary vessels with >75% stenosis tested on coronary angiography; (2) history of acute coronary syndrome; (3) coronary revascularization, either percutaneous transluminal coronary angioplasty or coronary artery bypass grafting).

The main exposure of interest was the use of ACE-Is and ARBs (including combinations with other antihypertensive drugs). Specifically, medication exposure was defined as having had active prescriptions of blood pressure (BP)-lowering medications (ACE-Is, ARBs, and other BP-lowering drugs) from at least 30 days before the date of admission. Other BP-lowering drugs included diuretics, beta-blockers, calcium channel blockers, and other antihypertensives, alone or in combination. Sacubitril/valsartan was categorized as an ARB. Even if the medications of interest were being withheld during hospitalization for any acute issues (i.e., hypotension, sepsis, acute kidney injury, and inability to take oral medications), these patients were still included based on their medication exposure. Investigators followed internal guidelines for the treatment of COVID-19 based on the clinical experience of the group. Our internal guidelines included the recommendation to modify the antihypertensive treatment (on clinical judgment) to achieve a systolic blood pressure <140 mmHg and a diastolic blood pressure <90 mmHg during the entire phase of hospitalization.

*Statistical analysis*. Analyses were performed using Stata, version 16 (StataCorp LP, College Station, TX, USA) and R version 2.9.2 (R Foundation for Statistical Computing, Vienna, Austria). We expressed continuous variables as mean ± standard deviation (SD) and the categorical variables as proportions.

We analyzed differences in proportions between groups using the χ2 test. Mean values of variables were compared using independent sample *t*-test or analysis of variance, when appropriate.

We evaluated the effect of prognostic factors on mortality using univariable and multivariable logistic regression analyses.

The odds ratios (ORs) from the univariable and multivariable analyses and their corresponding two-sided 95% confidence intervals (CIs) were derived from the regression coefficients in the logistic models. Survival curves were estimated using Kaplan–Meier product limit method and compared with the Mantel (log-rank) test.

We tested the prognostic impact of several variables, which proved a significant influence on mortality in this setting, and we modeled a multivariable model using the covariates that yielded statistical significance in the univariable analysis.

More specifically, we tested the prognostic impact of age (years) [15,16], history of diabetes (yes/no) [16], history of dyslipidemia (yes/no) [17], history of cardiac events (yes/no) [18], history of chronic obstructive pulmonary disease (yes/no) [19], renal function [16], hemoglobin levels (1 g/dL) [20], high-sensitivity C-reactive protein (CRP), troponin elevation, PaO_2_/FIO_2_ ratio, white blood cell and absolute lymphocyte count (1000/mcl) [17,18], oxygen saturation (%) at admission, history of neoplasm [19], and severe hypotension (yes/no) occurring during hospitalization and requiring inotropic support [17]. For continuous covariates, Youden index analysis was also used to identify the optimal cutoff value for the identification of patients at increased risk of death.

We used Akaike’s information criterion (AIC) and the Bayesian information criterion (BIC) to compare performance of different multivariable models. Analyses were performed using a significance level of α = 0.05 (2 sided).

## 3. Results

Overall, 566 hypertensive patients were included in the analysis (mean age 75 years; 54% males; 309 patients treated with RAS modifiers, including ACE-Is and ARBs). None of the patients were receiving a combination of ACE-Is and ARBs. Among patients treated with other BP-lowering drugs (see Materials and Methods), 50 were treated with monotherapy and 207 with combination therapy.

Baseline co-morbidities, specific in-hospital medications, and characteristics commonly used to define severe COVID-19 (age, severe hypotension, lymphocyte count, estimated glomerular filtration rate [eGFR], CRP, and PaO_2_/FIO_2_ ratio) were well balanced among different BP-lowering drug users (Table 1).

During hospitalization, 66 patients died. Among the RAS modifier users, 27 patients (9%) died in hospital, whereas among other BP-lowering drug users, 39 (15%) died. Thus, exposure to RAS modifiers was associated with a significant 46% reduction in the risk of in-hospital mortality when compared with other BP-lowering strategies (OR: 0.54, 95% CI: 0.32 to 0.90, *p* = 0.019).

**Figure 1:**
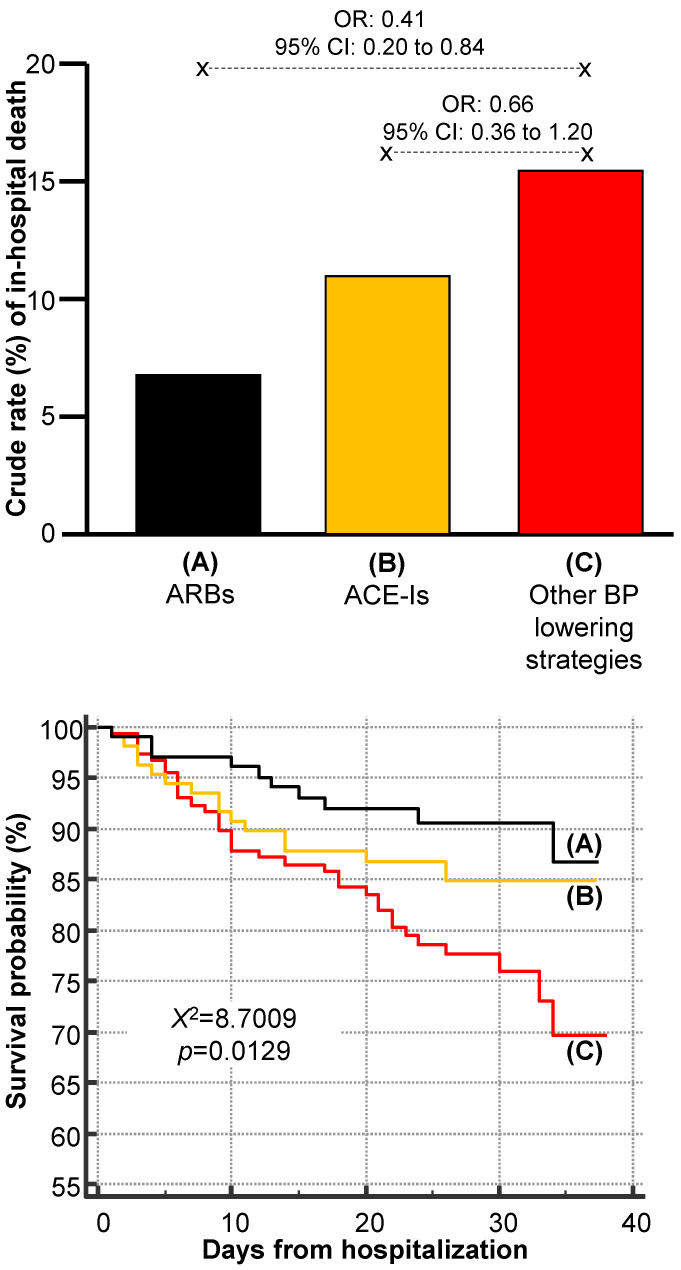
Risk of in-hospital death among hypertensive patients hospitalized for COVID-19 according to subgroups of antihypertensive therapy (upper panel). Survival curves (lower panel) were estimated using Kaplan–Meier product limit method and compared with the Mantel (log-rank) test. Legend: ACE-Is = ACE inhibitors; ARBs = angiotensin receptor blockers; BP = blood pressure; CI = confidence interval; OR = odds ratio.

We also evaluated the outcomes of hospitalized COVID-19 patients based on their exposure to ACE-Is, ARBs, and other BP-lowering drugs: 162 (29%) were ACE-Is users, 147 (26%) were ARBs users, and 257 (45%) used other antihypertensive medications. The rates of in-hospital mortality were 15%, 10%, and 7% for exposure to other BP-lowering drugs, ACE-Is, and RAS modifiers, respectively. Figure 1 depicts the crude rates of in-hospital death according to the subgroups of antihypertensive therapy. Estimating the risk of death according to logistic regression analysis, the group of ACE-Is users was not significantly associated with a reduced risk of in-hospital mortality when compared with patients treated with other BP-lowering strategies (OR: 0.66, 95% CI: 0.36 to 1.20, *p* = 0.172). Conversely, ARBs users showed a 59% lower risk of death (OR: 0.41, 95% CI: 0.20 to 0.84, *p* = 0.016, Figure 1—upper panel). Similar results were obtained using the Kaplan–Meier product limit method (Figure 1—lower panel).

**Figure 2:**
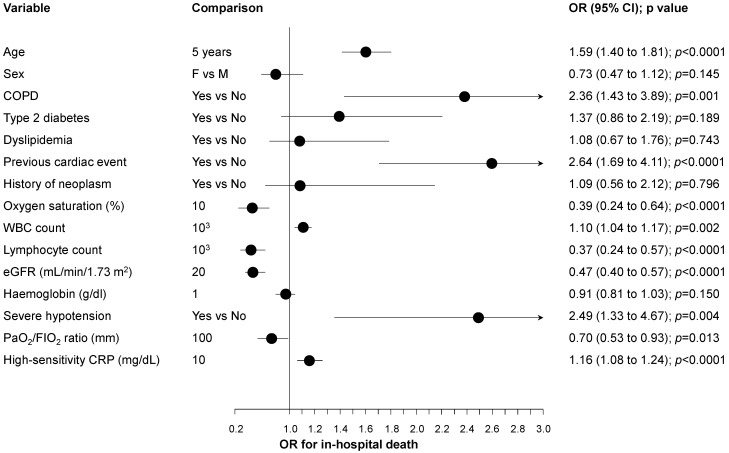
Results of univariable analyses exploring predictors of in-hospital death. Legend: COPD = chronic obstructive pulmonary disease; CRP = C-reactive protein; eGFR = estimated glomerular filtration rate using the Chronic Kidney Disease Epidemiology Collaboration (CKD-EPI) equation; WBC = white blood cell.

Among other covariates tested as predictors of in-hospital death (Figure 2), age, chronic obstructive pulmonary disease (COPD), previous cardiac events, a decreased oxygen saturation level recorded at admission, baseline high white blood cell count, severe hypotension occurring during hospitalization, lymphocytopenia at baseline, reduced eGFR at admission, reduced PaO_2_/FIO_2_ ratio, and increased high-sensitivity CRP were associated with an increased risk of death (all *p* < 0.05, Figure 2).

Using the categorization of continuous variables according to the optimal cutoff value as identified by the Youden index analysis (see Materials and Methods), the best informative multivariable model (baseline multivariable model, AIC = 358, BIC = 379, Table 2, upper panel) included age, severe hypotension, oxygen saturation, and lymphocyte count. 

When we added the exposure to ACE-Is or ARBs (other BP-lowering drugs used as reference) to the baseline model (Table 2, lower panel), ARBs were associated with a significant 63% lower risk of death (OR: 0.37, 95% CI: 0.17 to 0.80, *p* = 0.012), whereas ACE-Is were associated with a non-significant 27% lower risk of death (OR: 0.73, 95% CI: 0.38 to 1.40, *p* = 0.339) when compared with other BP-lowering strategies. 

Similar results were also obtained after the adjustment of other risk markers that proved statistical significance in the univariable analyses. To further characterize the effect of the severity of COVID-19 at admission as a prognostic modifier, we also evaluated the presence of myocardial injury and pulmonary involvement (see Materials and Methods). 

Overall, 99 patients (17%) showed cardiac involvement (high-sensitivity cardiac troponin elevation); the prevalence of troponin elevation was 19%, 17%, and 16% among patients treated with other BP-lowering drugs, ACE-Is, and ARBs, respectively (*p* = 0.649). The PaO_2_/FIO_2_ ratio was 318 mm, 316 mm, and 309 mm among patients treated with other BP-lowering drugs, ACE-Is (*p* = 0.990 vs. other BP-lowering drugs), and ARBs (*p* = 0.924 vs. other BP-lowering drugs and *p* = 0.973 vs. ACE-Is), respectively. When compared with the users of other BP-lowering strategies, patients exposed to ARBs showed a significant lower risk of in-hospital death even after adjustment for the significant effect of troponin elevation (*p* = 0.024) and the PaO_2_/FIO_2_ ratio (*p* = 0.012). More specifically, ARBs were associated with a significant 61% lower risk of death (OR: 0.39, 95% CI: 0.19 to 0.82, *p* = 0.012), whereas ACE-Is were associated with a non-significant 36% lower risk of death (OR: 0.64, 95% CI: 0.35 to 1.18, *p* = 0.151) when compared with other BP-lowering strategies and after adjustment for troponin elevation. Similar results were obtained after adjustment for the PaO_2_/FIO_2_ ratio (OR: 0.40, 95% CI: 0.17 to 0.95, *p* = 0.037 for ARBs vs. other BP-lowering drugs; OR: 0.76, 95% CI: 0.37 to 1.56, *p* = 0.456 for ACE-Is vs. other BP-lowering drugs). 

**Figure 3:**
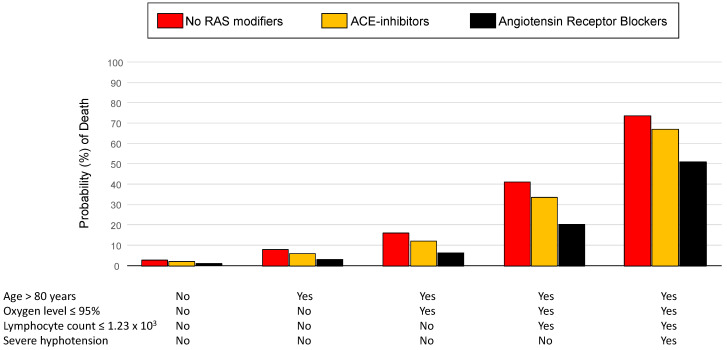
Effects of different blood pressure-lowering drugs on the probability (%) of in-hospital death according to different baseline risk strata (as identified by the presence of independent risk markers of prognosis).

The effects of the different BP-lowering drugs on the probability (%) of in-hospital death according to different baseline risk strata (as identified by the presence of risk factors included in the multivariable model) are depicted in Figure 3.

During hospitalization, the discontinuation of ACE-Is, ARBs, and other BP-lowering drugs was 17%, 18%, and 10%, respectively. Of note, the discontinuation of RAS modifiers during hospitalization did not exert a significant confounding impact (*p* = 0.515).

## 4. Discussion

The results of our study support the hypothesis that exposure to RAS modifiers reduces the risk of death during hospitalization for COVID-19. When splitting the users of RAS modifiers into ACE-Is and ARBs users, we found that exposure to ACE-Is was not significantly associated with a reduced risk of in-hospital mortality when compared with patients not treated with RAS modifiers. Conversely, ARBs users showed a decreased risk of death even after adjusting for a wide range of prognostic markers.

Some methodological aspects of our study deserve to be highlighted when compared to previous clinical studies. Available clinical studies often did not allow separate analyses of ACE-Is and ARBs, although it may be expected that these two different classes of antihypertensive drugs differently impact the prognosis of COVID-19 [4]. In our cohort, we had the opportunity to evaluate the different impacts of ACE-Is and ARBs on the management of COVID-19 hypertensive patients when compared with other BP-lowering drugs.

The large amount of evidence on the topic is mainly driven by retrospective studies [4,21,22,23,24,25,26,27,28,29]. Remarkably, our study was not a retrospective collection of clinical data in patients hospitalized for COVID-19 but rather a pre-designed protocol with subsequent prospective collection of data (see Materials and Methods).

Furthermore, exposure to different BP-lowering drugs was measured during a 30-day window before admission as an intention-to-treat analysis, and discontinuation was also evaluated during the entire hospital phase and evaluated as a possible confounding factor.

In other words, even if the medications of interest were being withheld during hospitalization for any acute issues, these patients were still included based on their medication exposure. Such an approach has the potential to avoid the bias related to the evidence that RAS modifiers tend to be continued in healthier patients and discontinued in patients with severe forms of the disease (including hypotension, low eGFR, and new kidney injury) [30].

Finally, we accounted for potential confounders by using a multivariable model adjusted for well-established prognostic factors, including age, severe hypotension, oxygen saturation, and lymphocyte count [9,15,16,17,18,19,20,31,32]. We also evaluated the effects of different BP-lowering drugs on the risk of in-hospital mortality among different baseline risk strata (as identified by the presence of different risk markers). As expected, the prognostic benefit of ARBs is magnified among patients with advanced age and with other laboratory features of increased risk (Figure 3).

In conclusion, the use of antihypertensive drugs and their potential impact on outcome in COVID-19 patients remain key points. Despite conflicting views in the literature, our results support the preferential use of ARBs.

Although the mechanisms explaining the potential benefits of RAS modifiers are still undetermined, some hypotheses have recently been proposed. There is growing evidence that the blunted ACE2 activity resulting from the reduced expression, downregulation, and dysfunction of these receptors after viral invasion and the resulting imbalance between angiotensin II and angiotensin_1–7_ may play an important role in conditioning inflammatory, thromboembolic, and hemodynamic processes in patients with COVID-19 [3,4,5,33,34].

**Figure 4:**
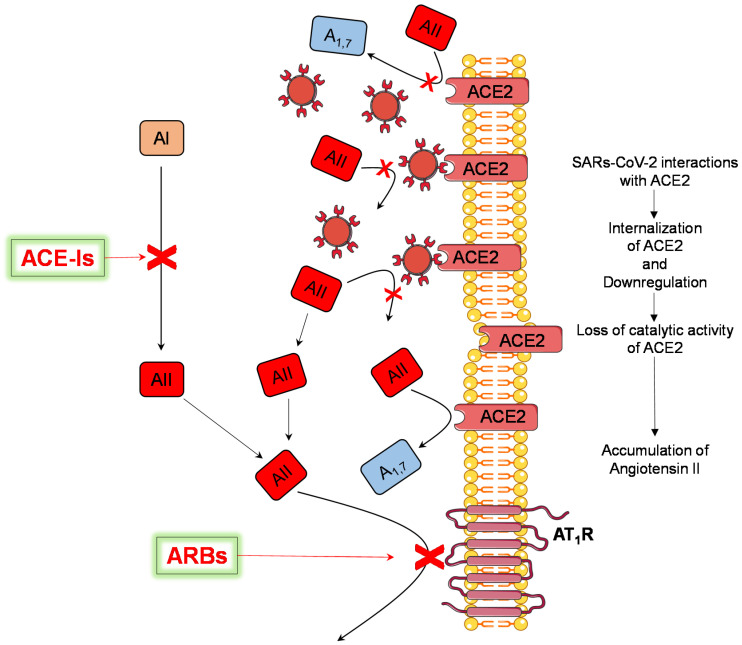
Potential mechanisms of pharmacological modulation of the renin–angiotensin system to reduce the deleterious effects of angiotensin II accumulation. AI = angiotensin I; AII = angiotensin II; A_1,7_ = angiotensin_1–7_; ACE2 = angiotensin-converting enzyme 2; ACE-Is = ACE inhibitors; ARBs = angiotensin receptor blockers; ATR1 = angiotensin II type 1 receptor.

Following this line of evidence, by enhancing ACE2 expression [35,36,37,38] and limiting the effects of unopposed angiotensin II on the heart, lungs, kidney, and vasculature, RAS-modifiers (and especially ARBs acting distally in the RAS to block the angiotensin II type 1 receptor selectively, Figure 4) have the potential to exert a better protective role in patients with COVID-19 when compared with other BP-lowering drugs [2,3,4,39,40,41,42,43,44,45]. Moreover, we can expect that ARBs exert these effects by both AT1 receptor blockade and angiotensin II type 2 (AT2) receptor stimulation [46,47]. Indeed, it has been reported that AT2 receptor stimulation antagonizes the effects of AT1 receptor stimulation in most tissues, promoting cardiovascular protection by the reduction of inflammation, oxidative stress, fibrosis, vascular remodeling, and vascular smooth muscle cell proliferation [46,47,48,49].

*Study limitations*. The present study should be interpreted within the context of its potential limitations. First, because the majority of enrolled patients were white, it may be difficult to extrapolate the results to different ethnic groups. Second, during data collection, some evidence was accrued on the prognostic significance of uncontrolled hypertension during hospitalization for COVID-19 [5,50]. In-hospital BP data were not routinely collected, but internal clinical guidelines were followed recommending that systolic BP be kept below 140 mmHg and diastolic BP below 90 mmHg. Third, the type, dosage, and duration of BP-lowering drugs and other cardiovascular concurrent medications, other than reported relevant laboratory results, electrocardiographic and echocardiographic findings, and specific causes of death were not collected. Finally, our study was not designed to evaluate the mechanisms of COVID-19 and the influence of BP-lowering drugs on the pathophysiology of the disease. Although the serum levels of angiotensin II and angiotensin_1–7_ were not measured in our study population, our results may indirectly support the hypothesis of an effect of RAS modifiers on angiotensin II accumulation observed during the acute phase of COVID-19. 

## Figures and Tables

**Table 1:** Main characteristics of patients included in the analysis.

Variable	Overall (*n* = 566)	ACE-Is (*n* = 162)	ARBs (*n* = 147)	Other BP-Lowering Drugs (*n* = 257)	*p*
Age (years)	75 ± 11	74 ± 12	76 ± 10	76 ± 11	0.060
Sex (male, %)	54	62	50	50	0.047
BMI (Kg/m^2^)	27.3 ± 5.7	27.6 ± 6.5	26.6 ± 4.2	27.5 ± 5.9	0.334
History					
COPD (%)	16	12	15	20	0.112
Type 2 diabetes (%)	31	30	31	31	0.936
Dyslipidemia (%)	32	28	36	33	0.330
Previous cardiac event (%)	29	30	22	32	0.144
Neoplasm (%)	11	11	15	9	0.139
Hospitalization					
Hydroxychloroquine (%)	58	58	60	56	0.715
Antiretroviral (%)	27	28	25	28	0.834
Macrolides (%)	32	28	38	30	0.298
Aspirin (%)	31	32	30	31	0.919
NSAIDs or glucocorticoids (%)	40	43	45	36	0.149
Oxygen level (%)	95 ± 3	95 ± 3	95 ± 3	95 ± 3	0.715
Severe hypotension	7	6	7	9	0.648
Haemoglobin (g/dL)	11.5 ± 1.8	11.7 ± 1.8	11.2 ± 1.7	11.5 ± 1.8	0.022
Lymphocyte count (× 10^3^)	1.51 ± 1.01	1.51 ± 0.66	1.63 ± 1.53	1.44 ± 0.76	0.238
eGFR (mL/min/1.73 m^2^)	72 ± 23	73 ± 24	72 ± 21	72 ± 25	0.818
K+ (ng/mL)	4.4 ± 0.6	4.4 ± 0.6	4.3 ± 0.5	4.3 ± 0.6	0.291
Troponin elevation (%)	17	17	16	19	0.649
PaO_2_/FIO_2_ ratio (mm)	315 ± 129	316 ± 110	309 ± 128	318 ± 141	0.852
High-sensitivity CRP (mg/dL)	11.5 ± 26.4	7.7 ± 18.1	15.5 ± 41.3	11.7 ± 26.4	0.068

Legend: BMI = body mass index; COPD = chronic obstructive pulmonary disease; CRP = C-reactive protein; eGFR = estimated glomerular filtration rate using the Chronic Kidney Disease Epidemiology Collaboration (CKD-EPI) equation; NSAIDs = non-steroidal anti-inflammatory drugs.

**Table 2:** Multivariable model exploring the impact of ACE inhibitors and angiotensin receptor blockers on the risk of in-hospital death (lower panel) when added to a baseline multivariable model identified according to information criteria (upper panel).

Variable	Comparison	OR	95% CI	*p*
**Baseline multivariable model**				
Age > 80 years	Yes vs. No	2.95	1.68 to 5.19	<0.0001
Severe hypotension	Yes vs. No	3.77	1.68 to 8.45	0.001
Oxygen saturation ≤ 95%	Yes vs. No	2.10	1.18 to 3.71	0.011
Lymphocyte count ≤ 1.23 × 10^3^	Yes vs. No	3.66	2.07 to 6.46	<0.0001
**Baseline multivariable model and antihypertensive drug subgroups**				
Age > 80 years	Yes vs. No	2.96	1.67 to 5.26	<0.0001
Severe hypotension	Yes vs. No	4.07	1.80 to 9.17	0.001
Oxygen saturation ≤ 95%	Yes vs. No	2.15	1.21 to 3.82	0.009
Lymphocyte count ≤ 1.23 × 10^3^	Yes vs. No	3.65	2.06 to 6.47	<0.0001
**BP-lowering drugs**				
ACE-Is	Other BP-lowering drugs	0.73	0.38 to 1.40	0.339
ARBs	Other BP-lowering drugs	0.37	0.17 to 0.80	0.012

Legend: BP = blood pressure; ACE-Is = ACE inhibitors; ARBs = angiotensin receptor blockers.

## Data Availability

The data underlying this article cannot be shared publicly due to the privacy of the individuals that participated in the study. The data will be shared on reasonable request to the corresponding author.
